# Definition of stereotactic body radiotherapy: Principles and practice for the treatment of stage I non-small cell lung cancer

**DOI:** 10.1007/s00066-013-0450-y

**Published:** 2013-09-21

**Authors:** M. Guckenberger, N. Andratschke, H. Alheit, R. Holy, C. Moustakis, U. Nestle, O. Sauer

**Affiliations:** 1 Department of Radiation Oncology, University of Würzburg, Josef-Schneider-Str. 11, 97080 Würzburg, Germany; 2 Department of Radiotherapy and Radiation Oncology, University of Rostock, Rostock, Germany; 3 Distler Radiation Oncology, Bautzen/Pirna, Germany; 4 Department of Radiation Oncology, RWTH Aachen University, Aachen, Germany; 5 Department of Radiation Oncology, University of Münster, Münster, Germany; 6 Department of Radiation Oncology, University of Freiburg, Freiburg, Germany

**Keywords:** Organs at risk, Survival, Toxicity, Patient positioning, Quality assurance, Risikoorgane, Überleben, Toxizität, Patientenpositionierung, Qualitätssicherung

## Abstract

This report from the Stereotactic Radiotherapy Working Group of the German Society of Radiation Oncology (Deutschen Gesellschaft für Radioonkologie, DEGRO) provides a definition of stereotactic body radiotherapy (SBRT) that agrees with that of other international societies. SBRT is defined as a method of external beam radiotherapy (EBRT) that accurately delivers a high irradiation dose to an extracranial target in one or few treatment fractions. Detailed recommendations concerning the principles and practice of SBRT for early stage non-small cell lung cancer (NSCLC) are given. These cover the entire treatment process; from patient selection, staging, treatment planning and delivery to follow-up. SBRT was identified as the method of choice when compared to best supportive care (BSC), conventionally fractionated radiotherapy and radiofrequency ablation. Based on current evidence, SBRT appears to be on a par with sublobar resection and is an effective treatment option in operable patients who refuse lobectomy.

## Abstract (de)

Die Arbeitsgruppe „Stereotaktische Radiotherapie“ der Deutschen Gesellschaft für Radioonkologie (DEGRO) erarbeitete eine Definition der Körperstereotaxie (SBRT), die sich an vorhandene internationale Definitionen anlehnt: Die SBRT ist eine Form der perkutanen Strahlentherapie, die mit hoher Präzision eine hohe Bestrahlungsdosis in einer oder wenigen Bestrahlungsfraktionen in einem extrakraniellen Zielvolumen appliziert. Zur Praxis der SBRT beim nichtkleinzelligen Bronchialkarzinom (NSCLC) im frühen Stadium werden detaillierte Empfehlungen gegeben, die den gesamten Ablauf der Behandlung von der Indikationsstellung, Staging, Behandlungsplanung und Applikation sowie Nachsorge umfassen. Die Körperstereotaxie wurde als Methode der Wahl im Vergleich zu Best Supportive Care, zur konventionell fraktionierten Strahlentherapie sowie zur Radiofrequenzablation identifiziert. Die Ergebnisse nach SBRT und sublobärer Resektion erscheinen auf aktueller Datenbasis ebenbürtig. Die SBRT ist die Methode der Wahl, wenn Patienten einen operativen Eingriff in Form der Lappenresektion ablehnen.

The principles and practice of stereotactic body radiotherapy (SBRT) were transferred from cranial stereotactic radiotherapy/radiosurgery in the mid-1990s by pioneering work at the Karolinska Hospital in Sweden [1]. This concept was quickly adopted and further developed in Japan [2] and Germany [3, 4]. At the beginning, SBRT was predominantly defined by frame-based stereotactic patient setup for accurate delivery of conformal dose distributions to extracranial targets in hypofractionated irradiation schemes. Nowadays however, a commonly accepted definition of SBRT does not exist. This is because frame-based stereotactic patient setup has been replaced by image guidance, which renders the term stereotactic misleading.

The Stereotactic Radiotherapy Working Group was asked by the German Society of Radiation Oncology (Deutschen Gesellschaft für Radioonkologie, DEGRO) to provide a definition of SBRT and to give an evidence-based review of current SBRT practice. This article will focus on the definition of SBRT, as well as the indications for and practice and outcome of SBRT for early stage non-small cell lung cancer (NSCLC).

## Definition of SBRT

National working groups in several different countries have reported their definitions of SBRT. The definitions of SBRT provided by the American Association of Physics in Medicine (AAPM) Task Group 101; the American Society for Therapeutic Radiology and Oncology and the American College of Radiology (ASTRO and ACR); the Canadian Association of Radiation Oncology—Stereotactic Body Radiotherapy (CARO-SBRT) and the National Radiotherapy Implementation Group of the UK [5, 6, 7, 8] all agree on the following items: SBRT is (1) a method of external beam radiotherapy (EBRT) that (2) accurately delivers a (3) high dose of irradiation in (4) one or few treatment fractions to an (5) extracranial target.

These essential components of the SBRT definition are specified in more detail below:

1. SBRT can be adequately performed with either traditional linear accelerators equipped with suitable image-guidance technology, accelerators specifically adapted for SBRT or dedicated delivery systems. Additionally, the principles of SBRT apply for both photon and particle therapy.
2. It is of fundamental importance that the entire SBRT workflow be systematically optimized and that appropriate quality assurance (QA) measures are implemented. From a clinical perspective, the term “accurate” covers disease staging; multidisciplinary discussion of the indications for SBRT; tumor site adjusted imaging with appropriate spatial and temporal resolution for target and organ at risk (OAR) definition; highly conformal treatment; image-guided patient setup; active or passive intrafraction motion management and follow-up (preferably at the treating institution). From a physics perspective, SBRT requires additional and more sophisticated QA procedures compared to conventional radiotherapy. These include system-specific end-to-end tests for both static and moving targets, as well as verification of the alignment of imaging and treatment isocenters prior to SBRT on a daily basis.
3. Radiation doses are at least equivalent to radical doses in conventional fractionation.
4. Radiation doses are delivered in few fractions (usually but not necessarily in a maximum of 10 fractions). Adjustment of single-fraction dose and total dose to volume and location of the target is essential.
5. The target is accurately localized using tumor site specific imaging modalities. Simultaneously, the target needs to be spatially separated from critical OARs, without diffuse infiltration into these. Only the macroscopic target and small, immediately adjacent volumes of potential microscopic spread are treated in SBRT.

 

The term “stereotactic” is still considered appropriate for SBRT, even if no frame-based patient setup using external stereotactic coordinates is performed; the external coordinates are replaced by the internal coordinates of the image guidance procedure. These images must either visualize and locate the target itself or surrogate anatomical structures/fiducial markers that are closely correlated to the target.

Stereotactic ablative radiotherapy (SABR) has been proposed by an international group of authors as an alternative to SBRT [9]. However, our opinion on the appropriateness of “ablative” differs because it does not accurately describe the radiobiology and clinical outcome of SBRT in all its variations. Techniques like radiofrequency ablation (RFA) are ablative irrespective of the normal or neoplastic nature of the tissue in the treated volume, which makes treatment of tumors abutting critical serial OARs impossible. In contrast, SBRT allows tumoricidal treatment of such targets while sparing the OARs through the differences in radiosensitivity of normal and cancerous tissue, as well as through fractionation effects.

## SBRT for early stage NSCLC

### Rationale for SBRT

After detection of NSCLC at an early stage (as is the case in 20–30 % of all patients), the best overall survival (OS) with cure rate in the majority of the patients is achieved by surgical lobectomy and systematic mediastinal lymph node dissection. However, surgical resection is not possible in a substantial proportion of patients—about 20 % of all patients with stage I NSCLC [10]—because of comorbidities or insufficient pulmonary function resulting from tobacco abuse and consequent chronic obstructive pulmonary disease (COPD). This fraction of inoperable patients is expected to further increase due to aging western populations: only 40 % of patients aged 75 years or older are treated surgically for stage I NSCLC in the Netherlands [11]. If no treatment is offered to these patients, cancer leads to death within few years despite its early stage [10].

In cases of contraindication for radical lobectomy, the standard treatment options are best supportive care (BSC), conventionally fractionated radiotherapy and sublobar resection. The therapy of choice depends on the patient’s performance status, age and comorbidities.

Based on the California Cancer Center registry, 7 % of all patients with stage I NSCLC are treated with BSC alone because their life expectancy is considered too short for curative treatment [10]. Among the elderly population, this proportion of untreated patients increases to 30 % [11, 12]. However, 5-year lung cancer specific survival rates are less than 20 % in untreated patients, indicating the need for a curative treatment option that is effective and simultaneously safe for this patient population.

Conventionally fractionated radiotherapy is an established curative treatment option for stage I NSCLC, with 3-year OS rates of about 30 % [13]. However, local tumor relapse is the most frequent cause of treatment failure after irradiation with conventionally fractionated doses of 60–66 Gy [13, 14]. Retrospective studies have demonstrated a dose–response relationship for local tumor control and disease specific survival in NSCLC [15, 16, 17], which clearly indicates the need for a radiotherapy methodology that enables a safe intensification of treatment.

Sublobar resection is practiced in borderline operable patients as an alternative to lobectomy. This technique spares lung tissue and preserves pulmonary function compared to lobectomy. However, the results of a randomized controlled trial [18] indicate that this is associated with inferior oncological outcome.

### Clinical outcome after SBRT for early stage NSCLC and implications for patient selection

#### SBRT vs. BSC in the elderly population and patients with severe pulmonary comorbidities

Two recent population-based analyses from the Netherlands [11, 12] and one from the US [19] demonstrated an improvement in OS for stage I NSCLC by the introduction of SBRT into the elderly patient population. OS was improved by shifting patients from BSC or conventionally fractionated radiotherapy to SBRT, treatment options which are both inferior to SBRT. Further studies showed that SBRT is safely practiced in patients with severe pulmonary comorbidities and very poor pretreatment pulmonary function and toxicity was not increased in these high-risk patient populations [20, 21]. Consequently, SBRT should be offered to all patients—irrespective of advanced age or pre-existing pulmonary comorbidities—unless their survival expectancy is very short. The SBRT characteristics of a noninvasive treatment delivered in a few fractions on an outpatient basis are expected to enhance acceptability in these patient cohorts.

#### SBRT vs. conventionally fractionated radiotherapy in medically inoperable patients

Several prospective phase II trials of SBRT in medically inoperable stage I NSCLC patients have reported local tumor control rates of 84–98 % [22, 23, 24, 25, 26, 27], demonstrating consistent results despite differences in SBRT methodology. This improved local tumor control compared to conventional radiotherapy [13] transfers into improved OS, as demonstrated in meta- [28] and population-based analyses [19]: 3-year OS is approximately 50 % after SBRT, with pretreatment comorbidities being a strong predictor for OS [29]. Based on these findings and the 1.2013 version of the National Comprehensive Cancer Network (NCCN) Guidelines for NSCLC [30], SBRT is today considered superior to conventionally fractionated radiotherapy and is the standard of care for medically inoperable patients.

#### SBRT vs. RFA

RFA has been introduced as a minimally invasive treatment option for stage I NSCLC. No study has performed a direct comparison between SBRT and RFA, but a recent literature review reported improved local tumor control, cancer specific survival and OS after SBRT compared to RFA [31]. Additionally, toxicity and 30-day mortality [32] were lower after SBRT, resulting in the conclusion that SBRT should be proposed as the initial nonsurgical treatment in high-risk patients.

#### SBRT vs. sublobar resection in high surgical risk patients

There are few studies directly comparing SBRT and sublobar resection for early stage NSCLC. Grills et al. [33] performed a single-institution comparison between SBRT and wedge resection. These authors reported improved local tumor control after SBRT, while no differences in regional control and cancer specific survival were observed. The previously cited US population-based analysis showed identical OS and cancer specific survival rates for SBRT and sublobar resection [19]: 30-day mortality rates were 0 and 1.2 % after SBRT and sublobar resection, respectively. Consequently, current data support both SBRT and sublobar resection as viable treatment alternatives for high surgical risk patients. The results of a currently recruiting American College of Surgeons Oncology Group randomized trial comparing SBRT and sublobar resection (ACOSOG-Z4099) are awaited.

#### SBRT vs. lobectomy for medically operable patients refusing surgical resection

The criteria for being fit enough to undergo lobectomy are described in national and international guidelines. Lobectomy is considered to be the treatment of choice for stage I NSCLC if these criteria are met. Few studies have reported outcome after SBRT where surgical resection was refused by the patients. Two Japanese and Dutch studies described excellent OS rates of 70 % after 5 years [34] and 85 % at 3 years [35], respectively. Two recent matched-pair analyses reported equivalent OS [36] and equivalent disease-free survival rates [37] after SBRT and lobectomy. Consequently, SBRT is a viable treatment option in the situation of surgical treatment being refused by the patient.

## Recommendations for clinical practice of SBRT in early stage NSCLC

### Clinical evaluation

Careful assessment of performance status is important to enable a sensible therapy concept. Perioperative morbidity is associated with older age (> 70 years) and the presence of comorbidities [38, 39]. Therefore, pulmonary function tests, as well as cardiac and performance status assessment are recommended before estimating the operative risk. As a corollary of this, the indication for SBRT should be discussed in an interdisciplinary tumor board.

#### Histopathological confirmation of disease

Histopathological confirmation of disease is recommended. Transbronchial biopsy or transthoracic needle aspiration are primary methods. Nevertheless, demonstration of malignancy is sometimes impossible or associated with an unacceptably high risk because of the medical and/or pulmonary comorbidities of the patient. In this case, radiological criteria of malignancy should be consulted.

Swensen et al. [40] described a prediction model to estimate the probability of malignancy in solitary pulmonary nodules (SPN) based on clinical and radiographic characteristics. This model was validated by Herder et al. [41]. Inclusion of fluorodeoxyglucose positron-emission tomography (FDG-PET) imaging might further improve the accuracy of the prediction model [42, 43]. If malignancy is highly likely based on the described criteria, immediate SBRT without histopathological confirmation is justified [44], as is also standard practice in thoracic surgery [45]. Repeated imaging to evaluate the growth pattern is an option in patients with a borderline risk of malignancy, but this might put the patient at risk of disease progression in the time interval [46].

#### Staging of disease

In accordance with other groups [47], the DEGRO working group recommends the following staging procedures prior to SBRT: a CT scan of the chest including the upper abdomen using intravenous contrast is mandatory. A whole body FDG-PET/CT scan should be performed in all cases. The added value of FDG-PET lies in the higher diagnostic accuracy for the detection of nodal metastases (negative predictive value: 90 %) [48, 49]; furthermore, distant metastases and clinically relevant second malignancies can be excluded. The PET/CT scan should not be older than 6 weeks. In case of pathological FDG uptake in mediastinal lymph nodes, further histopathological evaluation, e.g. by endoscopic ultrasound (EUS)- or endobronchial ultrasound (EBUS)-guided biopsy is mandatory. If the situation is still unclear, a mediastinoscopy may be necessary.

After exclusion of nodal metastases, SBRT is recommended for early stage NSCLC with a maximum tumor diameter ≤ 5 cm. Only limited data are available about the safety and efficacy of SBRT for lesions larger than 5 cm, where the risk of toxicity and occult systemic disease might be increased.

#### Imaging for target volume definition

Obviously, all images must be acquired in the treatment position. The planning CT scan should include the entire lung volume. Acquiring CT scans with a slice thickness of 2–3 mm is recommended. Use of intravenous contrast for CT scanning improves delineation of centrally located primary tumors. However, the use of synchronous intravenous contrast with four-dimensional CT (4D-CT) scans of the thorax is not established.

Due to the risk of artifacts and systematic errors introduced by the nonrepresentative nature of the captured breathing position, the use of a 4D-CT (also known as respiration-correlated CT) scan is strongly recommended for treatment planning and also allows for the evaluation of motion management strategies.

#### Target volume concept and motion management strategy

A gross tumor volume (GTV) should be defined based on CT findings in lung and soft tissue windows including all small spiculae. Most centers do not use clinical target volume (CTV) margins to account for microscopic tumor extensions. However, practices pertaining to CTV definition are inconsistent. Caution is therefore advised when applying this concept to SBRT target volume definition, as the applied safety margins may unnecessarily increase target volumes.

Integration of breathing-induced target motion into the target volume concept is a prerequisite for ensuring proper tumor targeting in individual patients. Clinical implementation depends on the respective motion management strategy, which in turn is required for SBRT.

Several different approaches can be applied and have already been implemented into routine practice: continuous irradiation during free breathing using (a) the internal target volume concept (ITV; most commonly practiced), (b) the mean target position concept [50] or (c) real-time tumor tracking. Irradiation in specific breathing phases is performed using (a) gated beam delivery, (b) voluntary breath holding or (c) breath holding using the Active Breathing Coordinator (Elekra, Stockholm, Sweden). Multiple planning studies reported reduced safety margins by the use of individually tailored patient motion compensation strategies compared to population-based margins.

Although patient-specific motion management is strongly recommended, it is important to note that no prospective trials have used advanced motion management strategies. Furthermore, no benefit of advanced motion management strategies like gating or tracking has been found for motion amplitudes < 10–15 mm [50, 51].

#### Dose, fractionation and prescription

Wherever possible, dose prescription and reporting should comply with the International Commission on Radiation Units (ICRU) Report 83. ICRU recommends prescribing to the median (D50), or alternatively, to the mean (D_mean_) dose. Based on experiences with cranial stereotactic radiotherapy, SBRT is practiced using inhomogeneous dose distributions within the planning target volume (PTV); maximum PTV doses range between 105 and 150 % of the prescribed PTV-encompassing dose. Effective doses to the GTV further vary according to the applied GTV-to-PTV safety margins and the size of the macroscopic tumor. We therefore recommend reporting minimum (D95 or D98) and maximum (D05 or D02) PTV doses—where DX is the minimum dose received by X% of the volume—in addition to the doses delivered to the GTV. Ideally, mean dose and the standard deviation of the mean dose should be reported as well as the afore mentioned dose levels. A sensible conformity index (CI) is the one proposed by Paddick [52]. If possible, this CI should be reported.

Normal tissue risk-adapted fractionation is highly recommended, with 1–10 delivered fractions depending on the size and central/peripheral location of the target [53].

Several groups independently demonstrated a clear dose–response relationship for local tumor control [54, 55, 56, 57]: a minimum biologically effective dose (BED; α/β ratio: 10 Gy) of > 100 Gy to the PTV) achieved local tumor control rates > 90 %. It could be demonstrated that this dose-dependent increase in local control translated into improved OS [54, 58]. A recent meta-analysis reported the best OS rates for medium to high SBRT doses of 83.2–146 Gy BED; OS was worse after SBRT with > 146 Gy BED, indicating a detrimental effect of excessively high doses [59].

Three fractions of 15 Gy with a dose maximum of 150 % was the preferred fractionation scheme in the German and Austrian patterns of care analysis and resulted in local tumor control rates of > 90 % [60]. This fractionation scheme of 3 × 15 Gy as D95 or D98 to the PTV with a D05 or D02 PTV dose of 150 % is therefore recommended for peripherally located NSCLC ≤ 5 cm in diameter. However, it has to be mentioned that this regimen was planned using type A (pencil beam) dose calculation algorithms in 80 % of cases, which might have overestimated the true dose by about 10 % [61]. Additionally, this recommendation is lower than the 3 × 18 Gy fractionation scheme that is used most frequently internationally.

Limited data are available concerning the safety and efficacy of SBRT for centrally located lesions [62]. Particularly where very high single fraction doses were applied, lethal outcomes have been observed [63]. Therefore, treatment of central lesions should preferably be performed within prospective trials such as the European Organisation for Research and Treatment of Cancer (EORTC) LungTECH trial. Until then, a recommended fractionation scheme for experienced centers is 8 × 7.5 Gy as D95 or D98 to the PTV; dose inhomogeneity within the PTV should be limited to 125 % [62].

#### OAR tolerance doses

It is of utmost importance to note that none of the published tolerance doses for SBRT have been formally validated. Nevertheless, as the toxicity after SBRT for peripheral lesions with the published OAR tolerance doses seems to be acceptable (grade ≥ II less than 10 %), adherence to such published protocols is strongly recommended (Tab. 1).

**Tab. 1:** Normal tissue constraints according to published major clinical studies. Radiation Therapy Oncology Group (RTOG) protocols can be found on the RTOG website at http://www.rtog.org/ClinicalTrials/ProtocolTable.aspx

Organ at risk	Single fraction (RTOG 0915)	Three fractions (RTOG 0618/1021)	Four fractions (RTOG 0915)	Five fractions (RTOG 0813)	Eight fractions (Haasbeck et al. 2011 [76])
**Trachea and large bronchus**	D_max_ 20.2 Gy	D_max_ 30 Gy	D_max_ 34.8 Gy 15.6 Gy < 4 cc	D_max_ 105 %^a^ 18 Gy < 5 cc^b^	D_max_ 44 Gy –
**Heart**	D_max_ 22 Gy 16 Gy < 15 cc	D_max_ 30 Gy –	D_max_ 34 Gy 28 Gy < 15 cc	D_max_ 105 %^a^ 32 Gy < 15 cc	–
**Esophagus**	D_max_ 15.4 Gy 11.9 Gy < 5 cc	D_max_ 25.2 Gy 17.7 G < 5 cc	D_max_ 30 Gy 18.8 G < 5 cc	D_max_ 105 %^a^ 27.5 Gy < 5 cc^b^	D_max_ 40 Gy –
**Brachial plexus**	D_max_ 17.5 Gy 14 Gy < 3 cc	D_max_ 24 Gy 20.4 Gy < 3 cc	D_max_ 27.2 Gy 23.6 Gy < 3 cc	D_max_ 32 Gy 30 Gy < 3 cc	D_max_ 36 Gy –
**Chest wall**	D_max_ 30 Gy 22 Gy < 1 cc	– 30 Gy < 30 cc 60 Gy < 3 cc [77, 78]	D_max_ 27.2 Gy 32 Gy < 1 cc	– 30 Gy < 30 cc 60 Gy < 3 cc [77, 78]	–
**Spinal cord**	D_max_ 14 Gy 10 Gy < 0.35 cc	D_max_ 18 Gy (RTOG 0236)	D_max_ 26 Gy 28.8 Gy < 0.35 cc	D_max_ 30 Gy 22.5 Gy < 0.25 cc	D_max_ 28 Gy

^a^PTV prescription^b^Volume constraint for non-adjacent wall*D*_*max*_ maximum dose.

Patients need to be informed about the following SBRT-specific toxicities: (1) Toxicities in up to 10 % of cases depending on tumor size and location: radiation-induced pneumonitis, rib fractures, chest wall pain. (2) Rarely observed toxicities: pleural and pericardial effusion, stricture of central airway structures, dyspnea, bleeding. (3) For more central locations, the low risk of life-threatening necrosis/fistula of neighboring organs such as esophagus, bronchi and large vessels, as well that of cardiac arrhythmias should be mentioned (depending on the exact tumor location).

#### Treatment planning

Photon energy should be below 10 MeV in order to avoid excess penumbra blurring. A leaf width of 5 mm at the isocenter is considered sufficiently small. The voxel size of the calculation grid should be 2 mm or less.

For dose calculation, heterogeneity correction and use of a type B dose calculation algorithm [64] is mandatory to achieve an accuracy of dose calculation exceeding 2 %.

All prospective trials used three-dimensional conformal radiotherapy (3D-CRT) for treatment planning. Intensity-modulated radiotherapy (IMRT) and advanced rotational techniques like volumetric-modulated arc therapy (VMAT) or RapidArc (Varian Medical Systems, Palo Alto, CA, USA) have the potential to increase conformity and homogeneity. The biological effect of these differences and the potential interplay of multileaf collimator (MLC) and tumor motion need to be considered. Flattening filter free irradiation will further reduce treatment delivery times.

#### Patient immobilization and setup

Customized patient immobilization was used in the majority of trials and is highly recommended to minimize intrafractional patient motion.

Internal shifts of the pulmonary target relative to the bony structures are the major source of positioning uncertainties [65, 66]. These effects cannot be countered by stereotactic setup or bone imaging. Therefore, daily pretreatment imaging is required for online correction of setup errors and base-line shifts. Imaging needs to visualize either the lung tumor directly or implanted markers that act as a surrogate for tumor position. Breathing-induced target motion must be fully integrated into the image-guided radiotherapy (IGRT) process. Post- and/or mid-SBRT imaging is recommended for QA, particularly in single fraction SBRT. Several methodologies for image guidance are commercially available (cone beam CT, in-room CT and orthogonal x-rays for fiducial control) and superiority of one method over the other has not been demonstrated. 

Interfractional tumor shifts might occur towards critical OARs and their correction by IGRT isocenter adjustment might result in increased OAR doses. Volumetric IGRT is required to visualize and quantify such effects. Methods for avoiding OAR overdosage are incorporation of safety margins for critical OARs (planning organ at risk volume, PRV, concept), repositioning or even replanning if critical thresholds are exceeded.

#### Follow-up

Clinical and radiological follow-up should be performed at the treating institution. CT imaging should be performed every 3 months for 2 years and every 6 months thereafter for another 3 years. This examination frequency has not been validated and should be adjusted to the patient’s performance status and suitability for salvage treatment.

Mass-like fibrosis within the high-dose region is observed after the majority of SBRT treatments [67, 68, 69, 70] and its differentiation from local failure is difficult. FDG-PET is recommended in cases where CT-based morphological differentiation of fibrosis and recurrence is not possible. However, FDG-PET results should be interpreted with caution within 6–9 months after SBRT due to the possibility of false-positive findings arising from inflammation processes [71]. Due to the risk of bleeding, bronchoscopic and/or transthoracic biopsies are only recommended if local recurrence is suspected and clinically relevant salvage treatment options are available.

## Physical and QA requirements for safe SBRT practice

It is recommended that SBRT be performed by a team consisting of a radiation oncologist, a medical physicist and technicians (radiographers, therapists). All members of the team should have attended dedicated SBRT teaching activities organized within the institution, by national or international societies or in an industrial setting.

Treatment should follow written standard operating procedures (SOPs), which are adjusted according to national regulations, the institution-specific equipment and training and education of the individual radiotherapy team members.

Comprehensive practical guidelines for SBRT are given in several international reports [5, 6, 7, 8, 47]. For Germany it is recommended to apply the appropriate norms defined by the Deutsche Institut für Normung (DIN). In this context the following reports are particularly relevant: DIN 6847-5, DIN 6858-1, DIN 6870-1, DIN 6873-5, DIN 6875-1 and DIN 6875-2.

Verification of the accuracy of the entire SBRT treatment chain is mandatory. This includes all imaging processes, the dose calculation engine, the MLC sequencing, the monitor unit calculation, the monitor calibration, patient positioning, the tracking or gating devices and so forth. Most steps are part of the regular commissioning and QA procedures.

It is paramount to verify that the radiation isocenter coincides with the mechanical isocenter—including couch rotation—and that the lasers are aligned to the radiation isocenter. The same is true for the imaging isocenter. End-to-end tests are recommended for overall uncertainty estimates. If a sufficiently precise independent and redundant localization is not used for every application, the alignment of the IGRT and the treatment isocenter should be tested daily.

## Radiobiological considerations of SBRT

Detailed understanding of the radiobiology of SBRT is still lacking. In contrast to conventional fractionated radiotherapy, there is no repopulation, reoxygenation or redistribution of tumor cells in single fraction SBRT. Even hypofractionated schedules still provide short overall treatment times and therefore at least accelerated repopulation has not to be considered. Additional effects of high fraction doses in SBRT may be vascular and [72] immune effects [73].

Although the linear quadratic (LQ) model is widely used in the community and has proven feasible for the description of SBRT outcome in several clinical reports, the applicability of this modality has been questioned because it has not been validated for very high single doses [74]. Most likely it is accurate up to single fraction doses of 15 Gy [75]. For higher fraction doses the model might overestimate the biological effectiveness, particularly for low α/β values. This may lead to underdosage of tumors with lower α/β values, such as prostate cancer, whereas it may be less critical for tumors with higher α/β values, such lung cancer. However, for normal tissues it should provide a more conservative approach. Alternative models have been proposed but are more complicated to use and have not been better validated using clinical data. In conclusion, the LQ model should be used, with caution, until more accurate models have been validated.

## Conclusion

This report provides a definition of SBRT that is agreement with that of other international societies. Detailed recommendations for principles and practice of SBRT for early stage NSCLC are given. These cover the entire treatment process; from patient selection, disease staging, treatment planning and delivery to follow-up.
